# 

**Published:** 1893-10

**Authors:** 


### CONTENTS.

### *EDITORIAL.*

PAGE
Reforming the Materia Medica, . . . . . . . . . . 481

### *MISCELLANEOUS.*

Potentiation Physiologically Proven. Prof. Dr. Gustav Jaeger. Translated by B. Fincké, M. D., . . . . . . . 483
Provings and Clinical Observations with High Potencies. Malcolm Macfarlan, M. D., . . . . . . . . . 488
Repertory of the Symptoms of the Ovaries. Cyrus M. Boger, M. D., 494
Cases from Practice. Dr. Hesse. Translated by A. McNeil, M. D., 503
Another Remedy for Asiatic Cholera. A. K. Crawford, M. D., . 505
How to Cure Rhus Poisoning. C. H. Evans, M. D., . . . . 509
The Resignation of Dr. Bell, . . . . . . . . . 510
Vaccinium and Variolinum. John Hall, M. D., . . . . . 510
World's Congress Transactions. Pemberton Dudley, M. D., . . 510
The Editorial in July Number. Edward Payson Small, M. D., . 511

### *BOOK NOTICES.*

Messrs. P. Blakiston & Co., . . . . . . . . . . 511
The Era Key to U. S. P., 1893, . . . . . . . . . 511

### *NOTES AND NOTICES.*

Dr. John Storer—Dr. A. W. Holcombe—The Purity of Cocoaine—Fun for Doctors, . . . . . . . . . . 512

